# PROMIS Fatigue short forms are reliable and valid in adults with rheumatoid arthritis

**DOI:** 10.1186/s41687-019-0105-6

**Published:** 2019-02-21

**Authors:** Clifton O. Bingham III, Anna Kristina Gutierrez, Alessandra Butanis, Vivian P. Bykerk, Jeffrey R. Curtis, Amye Leong, Anne Lyddiatt, W. Benjamin Nowell, Ana Maria Orbai, Susan J. Bartlett

**Affiliations:** 1 Johns Hopkins Medicine, Division of Rheumatology, Mason F Lord Center Tower, 5200 Eastern Ave #434A, Baltimore, MD 21224 USA; 2 Johns Hopkins Medicine, Division of Rheumatology, Mason F Lord Tower, 5200 Eastern Avenue, Rm 4100, Baltimore, MD 21224 USA; 3 Hospital for Special Surgery, Weill Cornell Medical College, 525 East 71st St, 7th floor, New York, NY 10021 USA; 4 Division of Rheumatology, University of Alabama at Birmingham, Birmingham, AL USA; 5 Healthy Motivation, Santa Barbara, CA USA; 6 Patient Partners, Ingersoll, ON Canada; 7 Global Healthy Living Foundation, Upper Nyack, NY USA; 8 Center for Health Outcomes Research, McGill University, 5252 de Maisonneuve West, #3D-57, Montreal, QC H4A 3S5 Canada

**Keywords:** Patient reported outcomes, Fatigue, Rheumatoid arthritis, Validation, PROMIS

## Abstract

**Background:**

Fatigue is prevalent and impactful in rheumatoid arthritis (RA). There is no standardized measure for its assessment nor data concerning the performance of PROMIS-Fatigue short forms (SFs) in people with RA. We evaluated the construct validity of 4-, 7-, and 8-item PROMIS-Fatigue SFs in RA patients across the range of disease activity.

**Methods:**

Adult RA patients were recruited from an online patient community and an observational cohort from three academic medical centers. Measures included PROMIS-Fatigue SFs, other PROMIS measures, and other patient reported outcomes including RAND-36 Vitality, Fatigue NRS, and patient global assessment of disease activity. Other measures from the observational cohort included 28-joint swollen and tender joints, physician global assessment, and the composite RA clinical disease activity index (CDAI).

**Results:**

Two-hundred online participants and 348 participants from the observational cohort were included. PROMIS Fatigue SF scores spanned the measurement continuum and correlated highly with each other (r’s ≥ 0.91) and other fatigue measures (r’s ≥ 0.85). PROMIS-Fatigue SF scores were highly and inversely associated with Physical Function and Participation (r’s − 0.77 to − 0.78), and moderately-highly and positively correlated with pain, sleep disturbance, anxiety, and depression (r’s 0.60 to 0.75). PROMIS-Fatigue SF scores showed dose-response relationships across fatigue severity descriptors and CDAI categories.

**Conclusions:**

These results provide robust evidence supporting the construct validity of the 4, 7, and 8-item PROMIS-Fatigue SFs. They capture fatigue across the spectrum of RA disease activity in diverse groups of individuals and should be considered for use as patient-centered assessments of disease control and treatment efficacy.

## Background

Fatigue in rheumatoid arthritis (RA) is a prevalent symptom that greatly impacts day-to-day function and quality of life [1, 2]. Fatigue experienced by patients with RA encompasses a broad spectrum, ranging from mild tiredness to an overwhelming, pervasive state of exhaustion that greatly impacts physical, emotional, and social function and quality of life. Higher levels of fatigue often track with other indicators of increasing RA disease activity [3]. Fatigue has been prioritized by RA patients as one of the most important symptoms for which they seek improvement with treatment [4]. Routine measurement of fatigue has been recommended for use in RA clinical trials, and when assessing remission of disease [5, 6].

Existing patient-reported outcome measures (PROMs) range from single item rating scales to multidimensional assessments, with no consensus on the optimal approach to fatigue assessment [6–8]. Many of the fatigue measures used in RA research (e.g., SF36-vitality, fatigue visual analog scale (VAS), Bristol Rheumatoid Arthritis Fatigue questionnaire (BRAF) have suboptimal psychometric properties, lack precision across the measurement continuum, or are poorly sensitive to change [7]. The NIH-developed Patient Reported Outcomes Measurement Information System (PROMIS®) fatigue measures have been shown to have robust psychometric properties across multiple chronic conditions although minimally studied in RA [9–12].

More than 90 items in the PROMIS-Fatigue item bank query the experience and impact of fatigue [9]. Computer-adaptive testing (CAT) using fatigue bank items provides reasonably reliable and valid estimates with efficiency and participant burden. While attractive for use in many settings, CATs require the availability of the internet, computers, or mobile technology (e.g., Smartphones), and algorithms for real-time administration and scoring. Moreover, the use of CATs in international clinical trials would require that the complete item bank has undergone translation and cultural validation into multiple languages. Thus in clinical trials and across health systems, where internet access to support CAT platforms may be limited, the use of fixed-item short forms (SFs) may be preferable to ensure reliable collection of data.

To measure fatigue over the past 7 days in adults, 4-, 7-, and 8- item PROMIS Fatigue SFs are available in English and other languages. Each SF asks about fatigue experience and impact. We administered the 8a short form which includes the 4 items on fatigue in the Profile-29; the 8-item adds 4 items. The 7-item SF contains different items. We have previously shown that PROMIS Fatigue SF items are easily understood and meaningful to people with RA [13].

There is limited information concerning the performance of PROMIS Fatigue SFs in RA. Here, we evaluate the reliability and validity and compare the performance of the SFs with each other, with legacy measures, and with other indicators of disease activity. We hypothesized PROMIS Fatigue scores would correlate highly with each other and be strongly and positively associated with worsening disease activity indices, pain, function, sleep and mood and reduced social participation.

## Methods

### Participants

We engaged participants from different locations to evaluate construct validity of PROMIS Fatigue SFs. We partnered with an online arthritis community (CreakyJoints.org) between July and September 2015 inviting them to participate (hereafter referred to as the “online” participants). They completed inflammatory arthritis questions from the Connective Tissue Screening Questionnaire [14] and an RA medication checklist (described elsewhere; (12) to exclude those with a personal or family history of psoriasis or psoriatic arthritis (PsA). The surveys were completed between July–September 2015. We also used the baseline data of 348 RA patients enrolled in an observational trial at academic arthritis centers in Baltimore, MD, New York City, NY, and Birmingham, AL.

### Fatigue measures

PROMIS SFs included the Fatigue 7a and 8a, Pain Interference 8a, Physical Function 20a, Depression 8a, Participation in Social Roles and Activities 8a, and PROMIS Adult Profile 29 [15], which also queries sleep disturbance and anxiety. The four RAND-36 [16] vitality items were added shortly after data collection began. Everyone rated average fatigue over the past 7 days using 5- and 11-point scales. Clinic patients also described their fatigue (*‘How would you rate your fatigue’*: none, mild, moderate, severe). Additional PROs included the patient global assessment of RA disease activity (0–100 NRS).

### Other outcomes

Among clinic patients, swollen and tender joint counts, physician global assessment, laboratory values, and Clinical Disease Activity Index (CDAI) scores from the visit were abstracted from patient charts. CDAI scores combine the patient and physician global assessments and joint counts to provide an index of disease activity; cut points define remission, low, moderate, and high disease activity.

### Statistical methods

We used the PROMIS Assessment Center Scoring Service to obtain item-response theory (IRT)-calculated scores. PROMIS scores are reported on a T-score metric (population mean of 50 and standard deviation of 10), with higher values representing more of the trait measured. We examined score distributions and compared descriptive statistics between clinic and online groups using t-tests or chi square. As participants from the three academic centers had similar characteristics, data were collapsed for subsequent analysis.

To evaluate reliability, we calculated Cronbach’s alpha within scales, and intra-class correlation coefficients (ICC; two-way mixed effects model where people were random and measures were fixed) to assess absolute agreement between measures. We used Bland-Altman plots to view the limits of agreement among measures and systematic differences. We considered values of 0.70 and 0.90 as evidence of acceptable group and individual reliability, respectively [17]. Convergent validity with two legacy fatigue measures (Rand Vitality and 10-point fatigue numeric rating scale) was assessed using Pearson’s r with correlations > 0.70 providing evidence of acceptable validity. We used ANOVA and trend tests to compare scores across disease activity groups. To examine floor and ceiling effects, we evaluated the proportion of participants at the lowest and highest scores (as provided in PROMIS Fatigue SF lookup tables); ≥ 15% of people at either end was considered evidence of an effect [18]. All analyses were performed in SPSS (v25), and a *p* < 0.05 was considered statistically significant.

## Results

### Participant characteristics

Participants were mostly female, white, and middle aged and had lived with RA on average for 14 years, with 13% having RA < 2 years (Table 1). Compared with clinic patients, online participants were significantly younger, more likely to be white and disabled due to their RA, and reported shorter disease duration, more years of education, and more active disease. Across clinical sites, characteristics were similar, except New York participants were more likely to report a higher level of education and urban residence and Birmingham participants reported greater disability due to RA.

**Table 1:** Characteristics of participants

Mean (SD) or %	All Clinic *n* = 348	Sites			Online *n* = 200	*P*-value
		NY *N* = 82	MD *N* = 217	AL *N* = 49		
Age (years)	57 (14)	56 (15)	56 (14)	60 (10)	51 (12)	<.001
Female (%)	81%	85%	80%	78%	84%	.422
Race^a^ (%)						
American Indian or Alaskan	3%	< 1%	3%	–	4%	
Asian	4%	9%	5%	–	1%	
Black or African American	10%	11%	13%	20%	4%	
Middle Eastern/North African	–	–	–	–	–	
Native Hawaiian/Pacific Islander	.2%	–	–	–	0.5%	
White	84%	71%	79%	80%	95%	
Other	1%	2%	2%	–	–	
Declined	1%	6%	< 1%	–	–	
Ethnicity						
Hispanic	7%	16%	4%	2%	6%	.714
Education > High school (%)	79%	91%	76%	74%	93%	<.001
Employment (%)						.011
Full time	36%	44%	35%	27%	32%	
Part time	8%	6%	9%	6%	8%	
Retired	23%	26%	25%	12%	15%	
Disabled due to RA	22%	14%	19%	49%	32%	
Other^b^	11%	9%	12%	6%	15%	
Urban	80%	90%	76%	80%	74%	.098
RA Characteristics						
RA duration (years)	14 (11)	13 (12)	13 (10)	17 (12)	10 (10)	<.001
Patient Global Disease Activity (0–100)	30 (27)	27 (23)	32 (28)	28 (27)	57 (23)	<.001

^a^Multiple categories selected by some individuals. **^**Between clinic and online samples

^b^Other includes unemployed, homemaker, students, disabled – not due to RA

### Fatigue scores

**Fig. 1:**
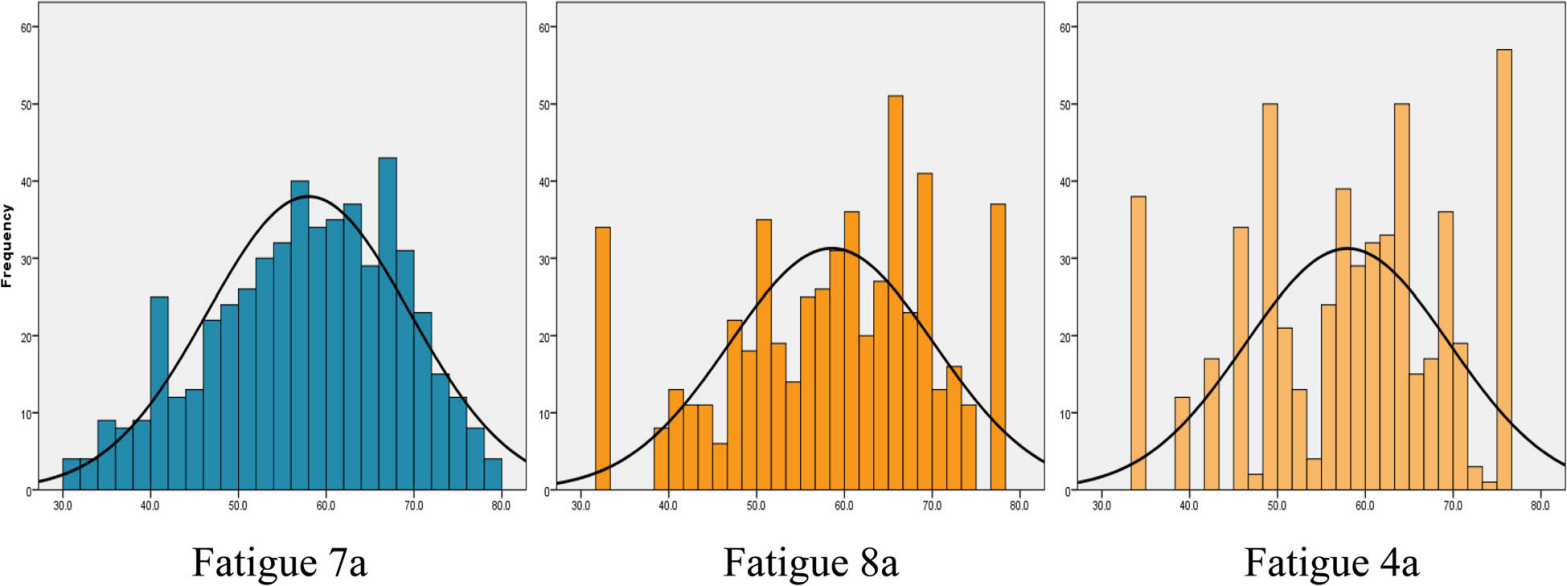
Distribution of PROMIS Fatigue 7a, 8a, and 4a scores

SF scores were relatively normally distributed, spanned a wide measurement continuum, and mean scores reflected significantly greater fatigue than the general population **(**Fig. 1**)**. Scores on the 7a spanned a larger range than 8a and 4a, and more closely approximated a normal distribution (Table 2). Scores on the other fatigue scales also spanned a wide range. As expected, PROMIS Fatigue SF scores were strongly correlated with each other (r’s ≥ 0.91) (Table 3) and with other fatigue measures (r’s ≥ 0.85).

**Table 2:** Scores on PROMIS Fatigue short forms and other patient reported outcomes

	N	Mean	SD	Median	25%	75%	Range	Min	Max
Fatigue									
PROMIS Fatigue 7a	548	58.0	11.5	58.7	50.1	66.4	53.3	29.4	82.7
PROMIS Fatigue 8a	548	58.6	11.6	60.1	50.5	67.3	44.6	33.1	77.7
PROMIS Fatigue 4a	546	58.0	11.6	58.9	48.6	66.6	42.1	33.7	75.8
Rand36 Vitality^a^	388	44.1	26.3	45.0	20.0	65.0	100.0	0.0	100.0
7-day fatigue (11 pt. NRS)	347	4.2	2.8	4.0	2.0	6.0	10.0	0.0	10.0
Fatigue Likert (1–5)	546	3.3	1.3	3.5	2.0	4.0	4.0	1.0	5.0
Other Measures									
PROMIS Physical Function 20a	546	38.7	9.4	37.3	31.8	44.1	43.7	18.8	62.5
PROMIS Pain Interference 8a	548	59.3	10.2	61.0	53.3	66.9	36.3	40.7	77.0
PROMIS Anxiety 4a	546	52.9	10.1	53.8	40.3	60.2	41.1	40.3	81.4
PROMIS Depression 8a	546	51.5	10.5	51.3	38.2	59.2	42.9	38.2	81.1
PROMIS Sleep Disturbance 4a	546	53.9	10.0	54.5	48.1	61.1	41.3	32.0	73.3
PROMIS Social Participation Ability 8a	548	44.8	10.3	43.9	37.1	51.7	39.5	25.9	65.4
Patient Global Disease Activity	547	22.7	29.6	25.0	2.0	49.0	100.0	0.0	100.0

^a^Collected only in clinic participants and added after data collection began

**Table 3:** Correlations among PROMIS fatigue 7a, 8a, 4a, Rand Vitality and 10-point fatigue scores

		Fatigue 8a	Fatigue 4a	Rand36	Fatigue NRS	7d Fatigue
Fatigue 7a	r	0.94	0.91	0.86	0.85	0.86
	N	548	546	388	347	546
Fatigue 8a	r		0.98	0.87	0.88	0.93
	N		549	388	351	546
Fatigue 4a	r			0.86	0.88	0.96
	N			387	348	544
Rand-36 Vitality	r				0.81	0.82
	N				344	387
Fatigue NRS (0–10)	r					0.87
	N					345

There was no evidence of floor or ceiling effects using PROMIS SF lookup tables. On the 7a, 1% had scores at the lowest (29.4) and highest (83.2) levels, based on PROMIS SF lookup tables. On the 8a, 6% were at the lowest (33.1) and 7% at the highest (77.7), while on the 4a 7% scores at the lowest (33.7) and 10% at the highest (75.8), well below the threshold of 15% for evidence of floor and ceiling effects.

### Reliability

**Fig. 2:**
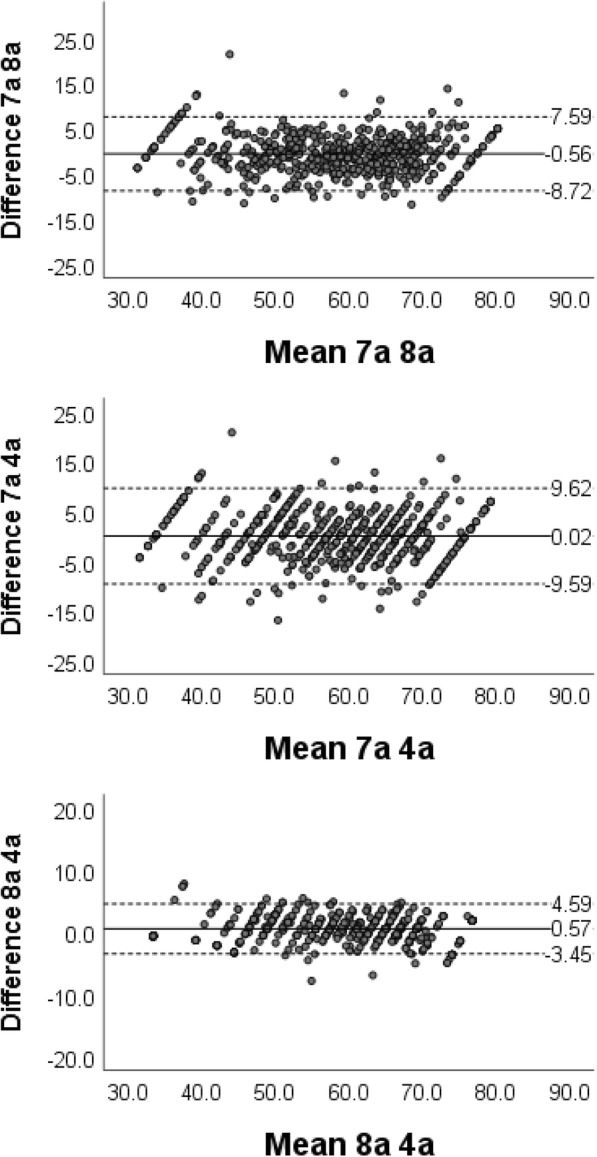
Bland-Altman plot showing correspondence between scores of PROMIS Fatigue short forms. Lines represent average difference and ± 1.96 standard deviation of the difference

Cronbach’s alpha showed evidence of adequate reliability at the individual level for 7a (0.96) and 8a (0.95), and at the group level for 4a (0.84). ICCs between scales for 7a and 8a were 0.93 (95% CI 0.92, 0.95), for 7a and 4a were 0.91 (95% CI 0.90 and 0.92), and for 8a and 4a were 0.98 (95% CI 0.98, 0.99). As Bland-Altman plots show (Fig. 2), mean differences between scores ranged from − 0.56 for the 7a and 8a to 0.02 for the 7a and 4a with no systematic differences evident. The 95% limits of agreement were 7.6 to − 8.7 for the 7a and 8a, and were somewhat larger for the 7a and 4a, and smaller for the 8a and 4a.

Mean standard errors for the 7a, 8a, and 4a were 2.8, 2.1, and 2.7. Across CDAI categories, mean SE were inversely related to disease activity level, and ranged from 2.7 to 3.1 for the 7a, 1.9 to 2.4 for the 8a, and 2.7 to 3.0 for the 4a suggesting adequate reliability at the individual level (i.e., >.90) for all levels of RA disease activity.

### Relationship of fatigue with other symptoms associated with RA disease activity

PROMIS-Fatigue SF scores were strongly and negatively associated with Physical Function and Participation (r’s − 0.77 to − 0.78), and moderately-highly and positively correlated with pain, sleep, anxiety, and depression (r’s 0.60 to 0.75) (Table 4). Associations were strongest between fatigue and other symptoms affected by worsening disease activity (pain, function, participation) [19]. As predicted, fatigue SFs scores were moderately and directly correlated with the patient assessments of disease activity (r’s 0.56–0.59), weakly to moderately and directly with tender joint counts, MD assessments of disease activity, and CDAI (r’s 0.32–0.50), but only minimally with observable characteristics such as swollen joint counts and laboratory markers of inflammation (r’s 0.15–0.22) (Table 4).

**Table 4:** Correlation of PROMIS fatigue short forms with other PROMIS measures and other clinical disease indicators of RA

PROMIS Fatigue		Physical Function	Participation	Pain Interference	Sleep Disturbance	Anxiety	Depression	TJC28	SJC28	MD	Patient	CDAI	ESR	CRP (mg/L)
7a	r	-0.78	-0.81	0.78	0.65	0.63	0.67	0.34	0.19	0.32	0.59	0.47	0.21*	0.17*
	N	546	548	548	546	546	546	345	346	343	547	340	219	254
8a	r	-0.78	-0.78	0.75	0.63	0.60	0.64	0.37	0.21	0.34	0.57	0.50	0.23	0.15**
	N	546	548	548	546	546	546	345	346	343	547	340	219	254
4a	r	-0.77	-0.77	0.75	0.65	0.59	0.63	0.37	0.22	0.35	0.56	0.50	0.22	0.15**
	N	546	546	546	546	546	546	343	344	341	545	338	219	252

All *p* < 0.001 except *p < .005 and **p < .02

**Fig. 3:**
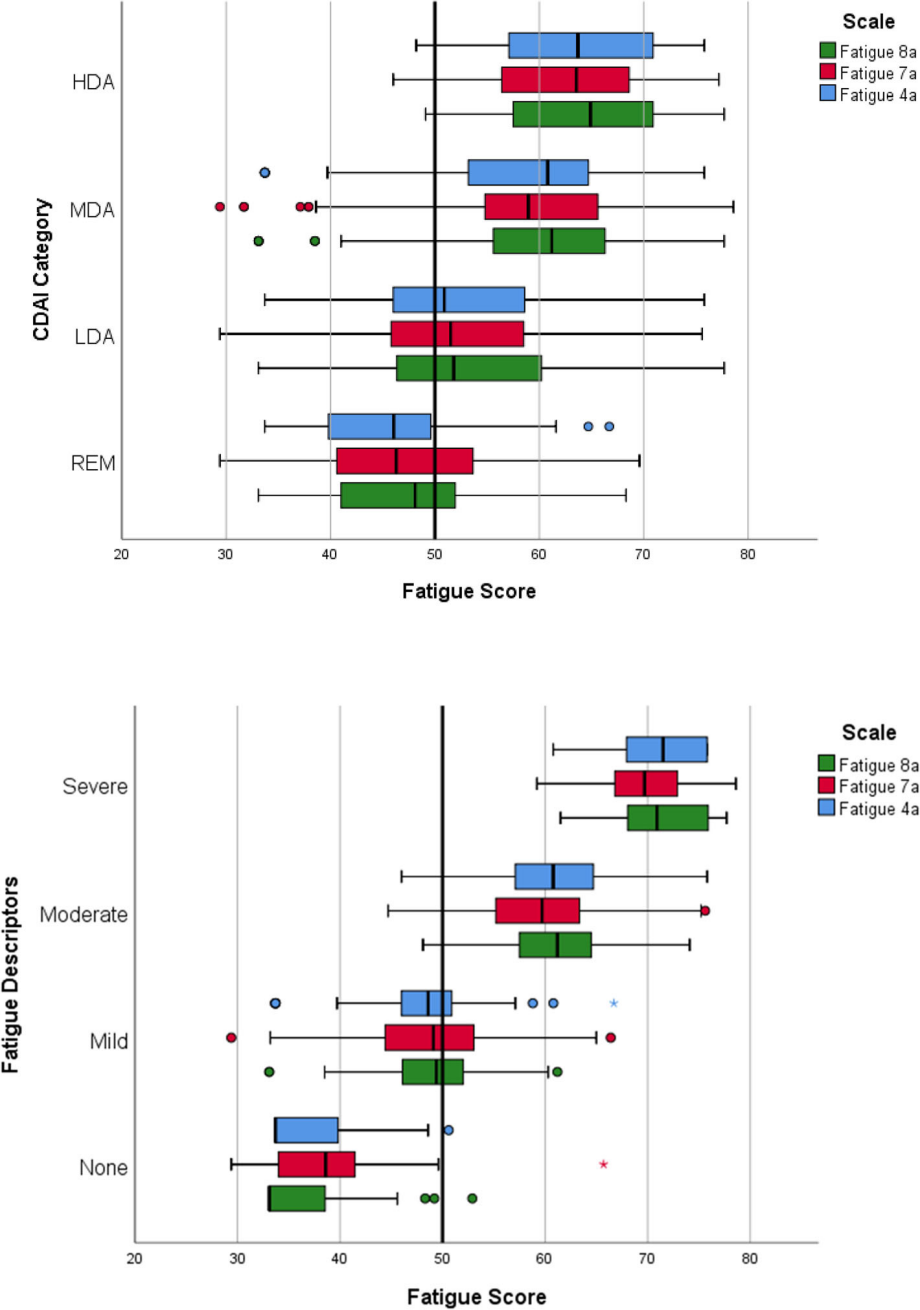
Box plots showing median and upper and lower interquartile ranges (IQR) of PROMIS Fatigue 4a, 7a, and 8a scores across Clinical Disease Activity Index levels and patient fatigue descriptors. Asterisks represent extreme scores > 1.5 IQR. REM = remission; LDA = low disease activity; MDA = moderate disease activity; HAD = high disease activity

Scores on all PROMIS fatigue SFs showed evidence of a dose-response relationship across the fatigue descriptors and CDAI categories (Table 5). The increase in mean PROMIS Fatigue SF scores across categories was at least ½-1 SD (i.e., 5-10 points). Within CDAI disease activity levels, considerable variability in fatigue scores was evident as shown in the box-plots in Fig. 3. Among those in remission, very few had fatigue scores >60 (1 SD worse than the US population mean); whereas, among those with high disease activity few had fatigue scores <50. Within descriptors of fatigue, there was also considerable variability though no patients with high fatigue had scores within the normal range (i.e., 45-55), and few patients reporting no fatigue had scores >50.

**Table 5:** Mean PROMIS fatigue scores by patient descriptors of symptom severity and indicators of RA disease activity

	N	Mean	SD	N	Mean	SD	N	Mean	SD	N	Mean	SD
Descriptors	None			Mild			Moderate			Severe		
Fatigue 7a	47	38.8	6.4	135	48.6	6.4	127	59.6	6.1	39	69.7	4.9
Fatigue 8a	47	36.3	5.1	135	49.2	5.3	127	61.1	4.9	39	71.4	4.7
Fatigue 4a	47	36.8	5.0	135	48.4	5.5	125	60.4	5.1	39	71.1	4.6
CDAI	Remission			Low			Moderate			High		
Fatigue 7a	80	46.3	8.4	132	52.0	9.6	94	58.4	10.7	34	62.7	8.4
Fatigue 8a	80	46.7	8.8	132	52.5	9.7	94	59.3	11.2	34	64.5	8.4
Fatigue 4a	80	46.3	8.7	131	51.9	9.8	93	58.6	11.0	34	63.9	8.5

Mean values in the same row are significantly different from each other at *p* < .05

## Discussion

Fatigue is ubiquitous in active RA, and can vary greatly in severity among individuals, and even within individuals from day-to-day. Among a large, geographically and racially diverse group of people whose RA symptoms varied from none (i.e., remission) to severe, fatigue levels also spanned the full measurement continuum. Scores on the three PROMIS SFs showed evidence of adequate reliability at the group level and correlated highly with each other, with legacy fatigue measures, and with other PROMs that reflect disease activity supporting construct validity. These results are consistent with our earlier work where we evaluated the performance of PROMIS-Fatigue CATs in relation to legacy fatigue scales and other PROMS, and our debriefing studies that showed the Fatigue items were easy to understand and relevant to people with RA [13].

Fatigue in RA has been hypothesized to result from several things including the greater physiological burden associated with high systemic inflammatory activity to increased difficulty moving due to swollen, painful joints. However, living with a chronic disease is also associated with greater depression and anxiety, sleep disturbance, and challenges maintaining participation in social roles and activities – each of which may also contribute to fatigue. Many fatigue measures query different aspects of fatigue ranging from simple global severity rating scales to multidimensional measures that ask about different ways people experience fatigue (e.g., cognitive difficulties, muscle weakness) to how it impacts daily activities (e.g., participation, work productivity, activities of daily living). Existing fatigue measures that are widely used vary in scope, length, domains, and statistical properties. PROMIS Fatigue SFs ask about the intensity of fatigue and its impact on day-to-day function. Our results suggest that each of the SF measures can discriminate among levels of fatigue across the full range of RA disease activity. PROMIS developers showed that Fatigue SF scores correlate highly (r’s > 0.95) with scores derived from CATs (see Fatigue Scoring Guide at healthmeasures.net) using the full item bank, with greatest precision of the SFs in the range from roughly 48 to 70, or in those with RA who describe their fatigue as ranging from none to severe.

Although it has been widely reported that fatigue increases with disease activity, additional mechanisms through which fatigue contributes to disability and suboptimal wellbeing associated with RA are not well understood. The Fatigue SF scores correlated most with other measures that are directly impacted by RA disease activity, including pain, function, and social participation (r’s > 0.7). Notably, though this sample included individuals with RA disease activity ranging from remission (i.e., no evidence of active disease) to high, mood and sleep disturbance were within the normal range in all but those with the highest levels of disease activity (data not shown), as we reported in an earlier study (11).

PROMIS-Fatigue SFs can quantify fatigue across the full continuum of RA disease activity. Scores increased significantly across CDAI levels by ≥0.5 SD (5 units), an amount that is widely recognized as clinically meaningful [20]. There was also evidence of dose-response relationships in SF scores as patients described the intensity of their fatigue worsening from ‘none’ to ‘severe’.

Mean scores were similar among SFs as shown by high ICCs, and on average differed by < 1 point even though the 7- and 8-item forms contain different items. These results from larger, more diverse samples of RA patients are similar to those we have previously obtained using PROMIS-Fatigue CATs [12]. However, the 95% limits of agreement between the 7a and 8a showed that there were often important differences at the individual level of up to 8–9 points (i.e., nearly 1 SD) which could result in misclassification.

The option to administer different subsets of test items via CAT or paper-based SFs reflects an important strength of using IRT-derived measures. While CATs result in better precision and efficiency, our results increase confidence that SFs containing 4-, 7-, or 8-items offer an acceptable alternative to measuring fatigue in RA when CAT administration of measures is not feasible. Notably, the 4-item version is contained in the PROMIS-29 Profile. The opportunity to choose from among SFs available with differing lengths is also a strength of PROMIS and IRT approaches to measurement optimization and may appeal to investigators who are hesitant to stop using existing familiar fatigue measures with which they are more familiar (and from which some PROMIS items may have been drawn).

Other benefits to using PROMIS SFs to measure fatigue include the reduced item number compared to some other legacy measures (e.g., FACIT-Fatigue, BRAF), thus reducing respondent burden. The availability of US general population norms facilitates comparisons of scores with those of age and sex-matched individuals. Finally, PROMIS-Fatigue SFs have been widely translated (7a-25 languages, 8a-29 languages, source: healthmeasures.net, accessed April 4, 2018), with additional efforts ongoing facilitating their use in multi-lingual populations and multinational studies.

Strengths of the study are the large diverse sample with a broad range of socio-demographic and RA characteristics. Our two-step screening approach increases confidence that the online sample included people with inflammatory arthritis. Among clinic participants, we triangulated fatigue scores with observations from clinicians and laboratory data. There are also limitations. Participants in the online sample had higher levels of reported symptoms and were self-selected for participation in the online survey, perhaps limiting generalizability of their results. However, in combination with the clinic-based sample with an average lower levels of symptoms we covered the range of disease activity across the studies. Data were drawn from the baseline visit of patients with established RA who were receiving care at tertiary arthritis clinics and enrolled in an observational trial. We recognize that comorbidities common to people with RA (e.g., diabetes, fibromyalgia, obstructive sleep apnea) could influence fatigue. However, our large sample size drawn from multiple sources likely helps overcome these concerns. As we used cross-sectional data, we could not examine responsiveness, and change in fatigue in relation to RA treatments or other intervention remain topics for future investigation. We did not evaluate structural validity; others have published results of extensive validity test of PROMIS Fatigue items across chronic diseases [9, 10, 21]. The evidence presented in our study came from IRT-based scoring of PROMIS scores through the Assessment Center rather than look-up tables. Finally, we examined performance of PROMIS Fatigue SFs in relation to a limited number of legacy fatigue measures.

## Conclusions

These results provide robust evidence supporting the construct validity of the three versions of PROMIS-Fatigue SFs that include 4, 7, and 8 items. Though CATs offer greater precision, the SFs can efficiently measure fatigue across the spectrum of RA disease activity in diverse groups of individuals and justify their consideration as one of the important patient-centered assessments that reflect disease control and treatment efficacy. Precise measures of fatigue using these PROMIS SFs can quantify fatigue associated with RA disease activity and its impact. Ultimately, these measures of fatigue can contribute to patient-centered discussions to help match individuals with optimal therapies based on health considerations that matter most to them.
